# Response of bacterial and fungal communities to high petroleum pollution in different soils

**DOI:** 10.1038/s41598-020-80631-4

**Published:** 2021-01-08

**Authors:** Polina Galitskaya, Liliya Biktasheva, Sergey Blagodatsky, Svetlana Selivanovskaya

**Affiliations:** 1 Institute of Environmental Sciences, Kazan Federal University, Kazan, 420008 Russia; 2 Institute of Plant Production and Agroecology in the Tropics and Subtropics, University of Hohenheim, 70599 Stuttgart, Germany; 3 Institute of Physico-Chemical and Biological Problems of Soil Science, Pushchino, 142290 Russia

**Keywords:** Environmental impact, Soil microbiology

## Abstract

Petroleum pollution of soils is a major environmental problem. Soil microorganisms can decompose a significant fraction of petroleum hydrocarbons in soil at low concentrations (1–5%). This characteristic can be used for soil remediation after oil pollution. Microbial community dynamics and functions are well studied in cases of moderate petroleum pollution, while cases with heavy soil pollution have received much less attention. We studied bacterial and fungal successions in three different soils with high petroleum contents (6 and 25%) in a laboratory experiment. The proportion of aliphatic and aromatic compounds decreased by 4–7% in samples with 6% pollution after 120 days of incubation but remained unchanged in samples with 25% hydrocarbons. The composition of the microbial community changed significantly in all cases. Oil pollution led to an increase in the relative abundance of bacteria such as *Actinobacteria* and the candidate *TM7* phylum (*Saccaribacteria*) and to a decrease in that of *Bacteroidetes*. The gene abundance (number of OTUs) of oil-degrading bacteria (*Rhodococcus* sp., candidate class *TM7-3* representative) became dominant in all soil samples, irrespective of the petroleum pollution level and soil type. The fungal communities in unpolluted soil samples differed more significantly than the bacterial communities. Nonmetric multidimensional scaling revealed that in the polluted soil, successions of fungal communities differed between soils, in contrast to bacterial communities. However, these successions showed similar trends: fungi capable of lignin and cellulose decomposition, e.g., from the genera *Fusarium* and *Mortierella*, were dominant during the incubation period.

## Introduction

Petroleum pollution in soils is currently considered one of the most serious environmental problems. This type of pollution decreases or fully destroys soil fertility, changes the elemental composition of soil and water cycles, leads to losses in the aesthetic value of ecosystems, causes secondary pollution of groundwater and air, and inhibits or eliminates soil organisms^1–5^.

Soil microorganisms, predominantly bacteria and fungi, play a major role in the decomposition of soil organic matter, synthesis of humus, cycling of nutrients, and promotion of plant growth. After their introduction into soil, hydrocarbons affect soil microorganisms directly or indirectly. Indirectly, petroleum leads to increased soil surface temperatures, changes in the content of soil organic matter, disturbances in the oxygen and water supply, and a decrease in nutrient availability. These changes, in turn, alter the structure and function of soil microbial communities^6–10^. Directly, petroleum hydrocarbons may inhibit soil community members, causing nonspecific membrane disturbances, damage to membrane functions, growth inhibition, and cell lysis^11–15^. Another direct effect of petroleum introduced into the soil is the growth stimulation of microbial populations that can decompose or tolerate hydrocarbons. These organisms are ubiquitous in soils, but their abundance and decomposition patterns are found to be dependent on soil physicochemical characteristics and soil genesis^16–20^.

Crude oil contains hundreds of hydrocarbons with different C chain lengths (from 1 to greater than 30), structures, and degrees of saturation. Usually, these hydrocarbons are grouped into aliphatics, aromatics, asphaltenes, and resins. The biodegradability of petroleum hydrocarbons decreases in the following order: n-alkanes > branched alkanes > low-molecular-weight aromatics > cyclic alkanes > high-molecular-weight aromatics and polycyclic aromatic compounds. Resins and asphaltenes are considered poorly biodegradable hydrocarbons^1,21–23^. Microorganisms use hydrocarbons as a sole carbon source or alter them to decrease their toxicity. The main pathways of microbial hydrocarbon decomposition occur under aerobic conditions; however, bacteria can also utilize hydrocarbons anaerobically^1,24–27^. Interestingly, bacteria have been demonstrated to decompose aliphatic and aromatic compounds, while fungi can also degrade polycyclic aromatics. Additionally, bacteria use specific metabolic pathways, such as those of alkane monooxygenase and dioxygenase, to decompose hydrocarbons, while fungi utilize different hydrocarbons in nonspecific enzyme complexes (e.g., cytochrome P450, lignin peroxidase, manganese peroxidase, and laccase) that enable them to decompose lignin and cellulose^28,29^.

In addition to the selection of populations that can decompose hydrocarbons occurring after petroleum pollution, microbial community successions in contaminated soils are determined by the initial soil microbial composition, as well as by the soil’s physicochemical properties and oil concentration^17,18,20,30^. The initial microbial composition is mainly influenced by the soil organic carbon content, pH, moisture and oxygen content, climate conditions, geographic position and soil genesis, and plants^19,31–39^. Particle size, clay content, and organic matter content are the main factors affecting the sorption and desorption of hydrocarbons in soil particles that, in turn, affect their toxicity and bioavailability^40–42^.

Efficient microbial decomposition of petroleum hydrocarbons occurs in the soil at a relatively low initial content of petroleum (up to 5%); therefore, the dynamics of microbial communities, especially those of bacteria, have been well studied for such cases^43–47^. However, less is known about the microbial communities of soils with high petroleum contents because they are considered to be eliminated or strongly inhibited due to their low activity. However, information about such communities is important to understand the potential of restoring soil ecosystems, e.g., after long time periods or with additional methods of remediation. Poor information is available about the dynamics of fungal and bacterial communities in petroleum-polluted soils with a focus on specific genes enabling different metabolic pathways of hydrocarbon decomposition. Few data are available concerning the similarities and dissimilarities of microbial community successions in different soils after oil disturbance of the soil environments. Since bacteria and fungi are the main agents that enable improvement of soil quality after petroleum pollution, in the case of both self-restoration and bioremediation^23,48^, the missing information has both fundamental and practical significance.

In this study, laboratory modeling of the self-restoration of three petroleum-polluted soil samples with different physicochemical characteristics was conducted over 120 days. Fungal and bacterial community structures, as well as bacterial catabolic gene abundance, were analyzed along with the hydrocarbon content in soils. We hypothesized that at high petroleum concentrations, the main soil physicochemical characteristics play a lesser role than hydrocarbons in the successions occurring in the soil microbial communities. Furthermore, it was hypothesized that bacterial communities are altered more significantly than fungal communities because of the presence of specific and absence of nonspecific metabolic pathways for hydrocarbon decomposition.

## Results

### Hydrocarbon content and composition

The petroleum hydrocarbon content revealed using the selective extraction method during the incubation was close to the amount of oil applied to the soil as an artificial contaminant (60 and 250 g kg^−1^ in the l-samples and u-samples, respectively). The estimated concentrations in different soils ranged between 48 and 53 g kg^−1^ for the low amendment rate (l-samples) and between 191 and 203 g kg^−1^ for the high amendment rate (u-samples, Supplementary Table S1). The lowest hydrocarbon content was revealed for chernozem (C) samples immediately after spiking with either oil concentration. The total petroleum hydrocarbon content did not change significantly after 120 days of incubation. However, the fractional composition of extracted petroleum was altered, especially in u-samples amended with the high amount of hydrocarbons. Thus, the proportion of aliphatic and aromatic compounds decreased by 4–7%, while the proportion of asphaltenes and resins was maintained at the initial level or elevated (up to 10%).

**Figure 1:**
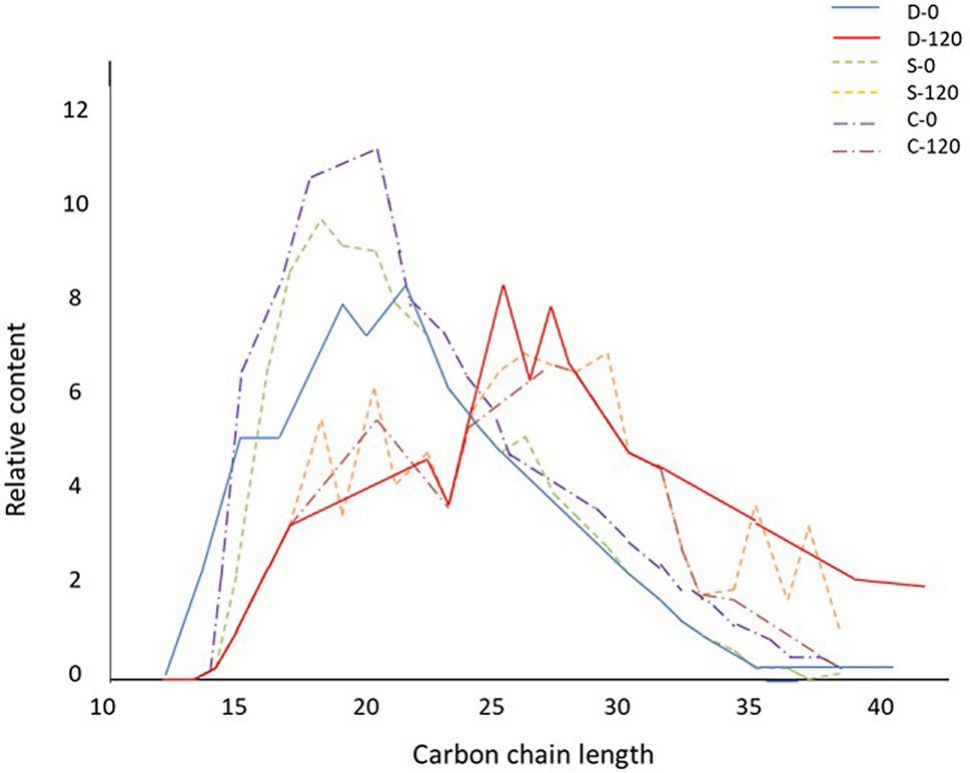
Relative content of n-alkanes with different C-chain lengths in the D, S, and C soil samples contaminated with 6% (l) and 25% (u) petroleum.

**Figure 2:**
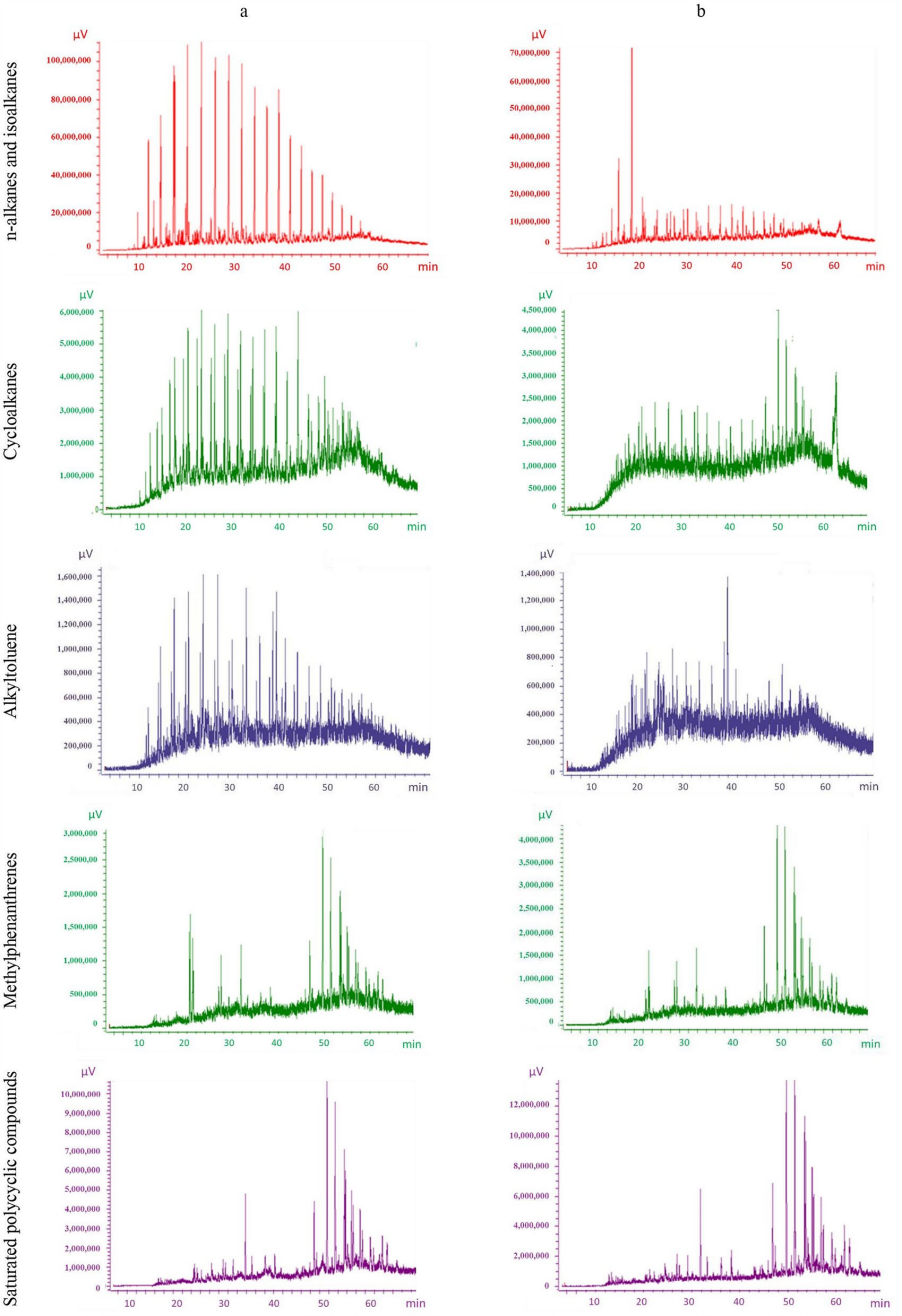
Composition of the different groups of hydrocarbons in the S soil sample with 6% crude oil at the beginning (**a**) and end of incubation (**b**).

In addition, the composition within the aliphatic and aromatic hydrocarbon groups was altered during the 120 days of the experiment. Thus, the proportion of short-chain alkanes decreased during incubation, while that of long-chain alkanes increased in all three soil types (Fig. 1). The composition of cycloalkanes and alkyl toluenes changed along with that of n-alkanes during the incubation of oil-polluted soils, while the composition of methyl phenanthrenes and polycyclic saturates did not change significantly, as seen from GC–MS chromatograms for the Luvisol sample (Sl sample, Fig. 2).

It was assumed that the observed changes in the fractional composition of hydrocarbons were caused by microbial biodegradation. The composition of soil microbial communities and their ability to utilize or transform hydrocarbons, in turn, may have been influenced by direct and indirect effects of the added oil. Therefore, we analyzed the composition of the bacterial and fungal communities in the initial and polluted soils.

### Bacterial and fungal counts in unpolluted and oil-polluted soil samples as revealed by qPCR

The unpolluted soils at the beginning of the experiment had from 1.3 × 10^7^ (sample C0) to 1.4 × 10^7^ (sample D0) bacterial gene copies g^−1^ and from 5.1 × 10^4^ (sample C0) to 2.3 × 10^5^ (sample D0) fungal gene copies g^−1^. At the end of the experiment, the bacterial count ranged from 9.0 × 10^6^ (sample D0-120) to 1.1 × 10^7^ (sample C0-120), and that of fungi ranged from 5.6 × 10^4^ (sample C0-120) to 1.3 × 10^5^ (sample D0-120) (Table 1). Pollution caused a slight decrease in the bacterial gene abundance in all of the samples on the 3rd day of incubation, by 1.3–4.1-fold, and no change was observed in the fungal gene abundance. However, these changes were in the range of natural fluctuations in microbial abundance in unpolluted soils. At the end of the experiment, no significant difference was found between the microbial abundance in the polluted and unpolluted samples.

**Table 1:** Copy numbers of bacterial 16S rRNA and fungal 18S rRNA genes in unpolluted and oil-polluted soils.

Sample	Bacterial 16S rRNA gene copy number, g-1				Fungal 18S rRNA gene copy number, g-1			
	3	30	60	120	3	30	60	120
D0	(1.4 ± 0.0) × 10^7^	NA	NA	(9.0 ± 0.6) × 10^6^	(2.3 ± 0.1) × 10^5^	NA	NA	(1.3 ± 0.0) × 10^5^
S0	(1.3 ± 0.0) × 10^7^	NA	NA	(9.3 ± 0.5) × 10^6^	(1.3 ± 0.0) × 10^5^	NA	NA	(7.5 ± 0.4) × 10^4^
C0	(1.3 ± 0.0) × 10^7^	NA	NA	(1.1 ± 0.0) × 10^7^	(5.1 ± 0.1) × 10^4^	NA	NA	(5.6 ± 0.3) × 10^4^
Dl	(1.3 ± 0.0) × 10^7^	(1.5 ± 0.0) × 10^7^	(2.1 ± 0.1) × 10^7^	(7.1 ± 0.4) × 10^6^	(1.9 ± 0.0) × 10^5^	(2.1 ± 0.1) × 10^5^	(2.1 ± 0.1) × 10^5^	(9.9 ± 0.8) × 10^4^
Du	(8.8 ± 0.7) × 10^6^	(1.2 ± 0.0) × 10^7^	(4.9 ± 0.3) × 10^6^	(9.9 ± 0.8) × 10^6^	(3.6 ± 0.2) × 10^5^	(2.0 ± 0.1) × 10^5^	(2.0 ± 0.1) × 10^5^	(4.7 ± 0.3) × 10^5^
Sl	(1.0 ± 0.0) × 10^7^	(1.3 ± 0.0) × 10^7^	(1.6 ± 0.0) × 10^7^	(1.1 ± 0.0) × 10^7^	(1.5 ± 0.0) × 10^5^	(1.5 ± 0.0) × 10^5^	(1.5 ± 0.0) × 10^5^	(1.5 ± 0.0) × 10^5^
Su	(7.6 ± 0.5) × 10^6^	(1.1 ± 0.0) × 10^7^	(2.4 ± 0.1) × 10^6^	(3.9 ± 0.1) × 10^6^	(1.3 ± 0.0) × 10^5^	(5.3 ± 0.4) × 10^5^	(5.3 ± 0.4) × 10^5^	(6.2 ± 0.5) × 10^4^
Cl	(3.0 ± 0.1) × 10^6^	(4.9 ± 0.2) × 10^6^	(8.3 ± 0.5) × 10^6^	(8.6 ± 0.6) × 10^6^	(3.4 ± 0.1) × 10^4^	(9.9 ± 0.8) × 10^4^	(9.9 ± 0.1) × 10^4^	(5.8 ± 0.5) × 10^4^
Cu	(3.2 ± 0.1) × 10^6^	(5.2 ± 0.4) × 10^6^	(3.0 ± 0.2) × 10^6^	(6.2 ± 0.4) × 10^6^	(2.6 ± 0.1) × 10^4^	(7.8 ± 0.6) × 10^4^	(7.8 ± 0.1) × 10^4^	(3.8 ± 0.4) × 10^4^

### Composition of bacterial and fungal communities in unpolluted soils

The composition of bacterial and fungal communities was estimated from 16S rRNA and ITS gene fragment sequencing using Illumina MiSeq. Among the unpolluted and polluted soil samples, 2,209,983 bacterial and 488,723 fungal sequences were estimated after the exclusion of 144,418 and 27,613 chimeras, respectively. The bacterial sequences corresponded to 691 unique OTUs—608 were found at least once in at least one of the unpolluted samples (D0, C0, S0 at the beginning and end of the experiment), and 83 were only found in polluted samples. The fungal sequences corresponded to 640 unique OTUs—286 were found at least once in the unpolluted soils, and 354 were found in polluted soils (Supplementary Tables S2, S3).

**Figure 3:**
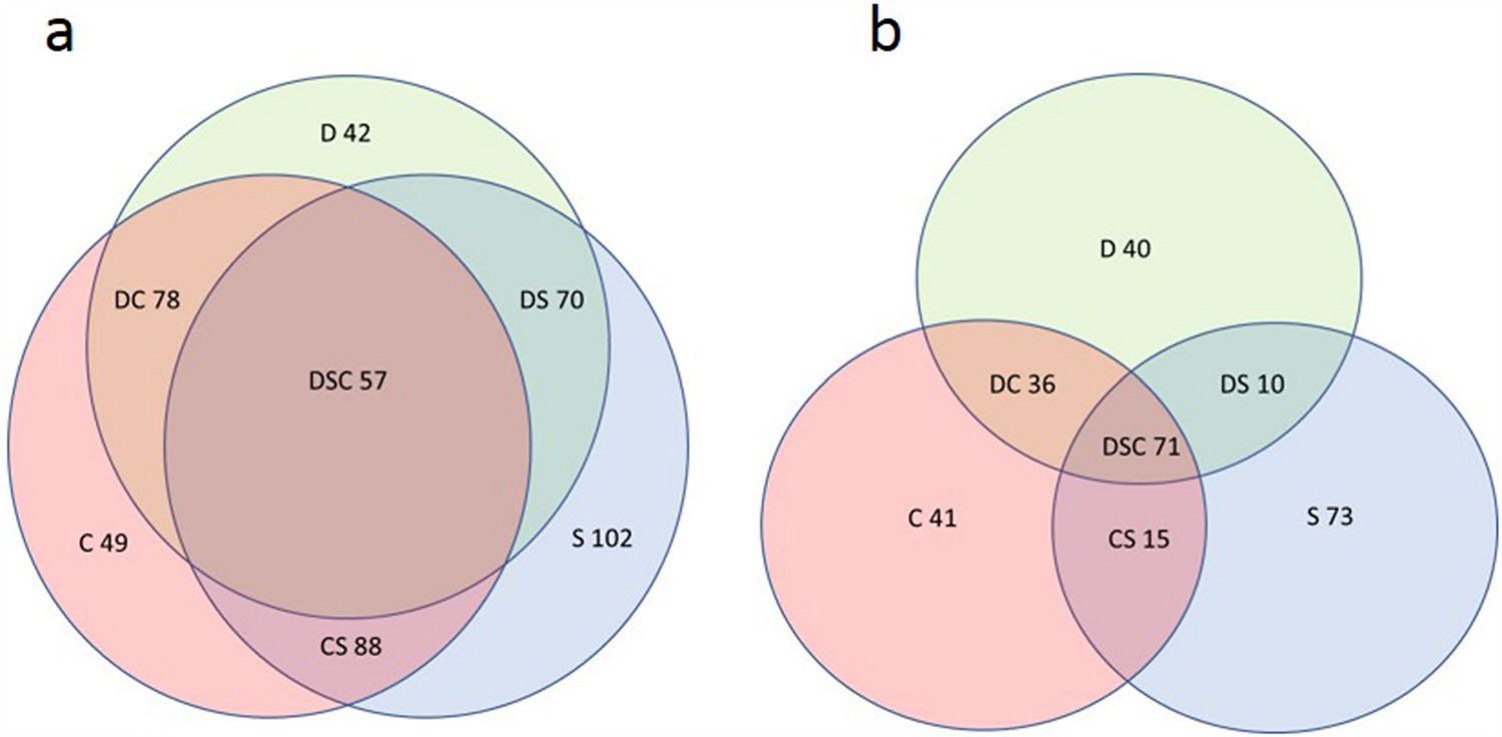
Venn diagram of the number of unique and common OTUs in bacterial (**a**) and fungal (**b**) soil communities.

Of the 608 bacterial OTUs found in the unpolluted soils, 485 were revealed in the D sample, 451 in the S sample, and 518 in the C sample. Thus, 375 OTUs (from 72 to 83%) were common for all the samples: 18–48 OTUs were common for two of three samples, and 28–65 OTUs were unique for the samples. Among 286 fungal OTUs found in unpolluted soils, only 71 were common for all three soil types, representing less than half of the total amount of OTUs in each sample—157, 169, and 163 for the D, S, and C samples, respectively. Additionally, 10–30 OTUs were found in two of three samples, and 40–73 OTUs were unique for each sample. As presented in the Venn diagrams (Fig. 3), the bacterial communities showed stronger overlap than the fungal communities in terms of their OTU pool.

The bacterial communities in all the soil types were close in terms of the abundances of different OTUs. Thus, the predominant phyla in all the samples were *Actinobacteria* (20–28%) and *Proteobacteria* (30–36%). Additionally, the dominating OTUs were similar in all three samples at the beginning and end of the experiment.

The most abundant OTUs were *DAD101* sp. from the phylum *Verrucomicrobia* (3–10%), representatives of the *Bradyrhizobiaceae* family (2–5%), *Rhodoplanes* sp. (5–8%), *Kaistobacter* sp. (1–7%) from the *Proteobacteria* phylum, representatives of the *iii1-15* order from the *Acidobacteria* phylum (5–6%), members of the *Gaiellaceae* family from the *Actinobacteria* phylum (3–9%), and the *Chitinophagaceae* family from the *Bacteroidetes* phylum (2–5%). All of the dominant OTUs were present in all communities, indicating that the composition of bacterial communities in the three investigated soil samples was similar. Additionally, no OTUs vastly exceeded others in the communities, indicating that the bacterial communities were developed and had relatively high biodiversity.

*Actinomycota* was a predominating phylum with 45–70% abundance in the studied samples, followed by *Basidiomycota* and *Mucoromycota.* In contrast to bacterial OTUs, there were more unique OTUs in the soil samples than ubiquitous OTUS. Among ubiquitous OTUs, OTUs from the genera *Fusarium* (2–64%), *Sagenomella* (1–15%), *Mortierella* (1–12%), *Trichoderma* (1–3%), and *Tetracladium* (1–2%), as well as those from the order *Pleosporales* (1–4%) and class *Tremellomycetes* (1–17%), were revealed. The abundance of the other dominating OTUs ranged between 1 and 17%.

### Bacterial communities in oil-polluted soils

**Figure 4:**
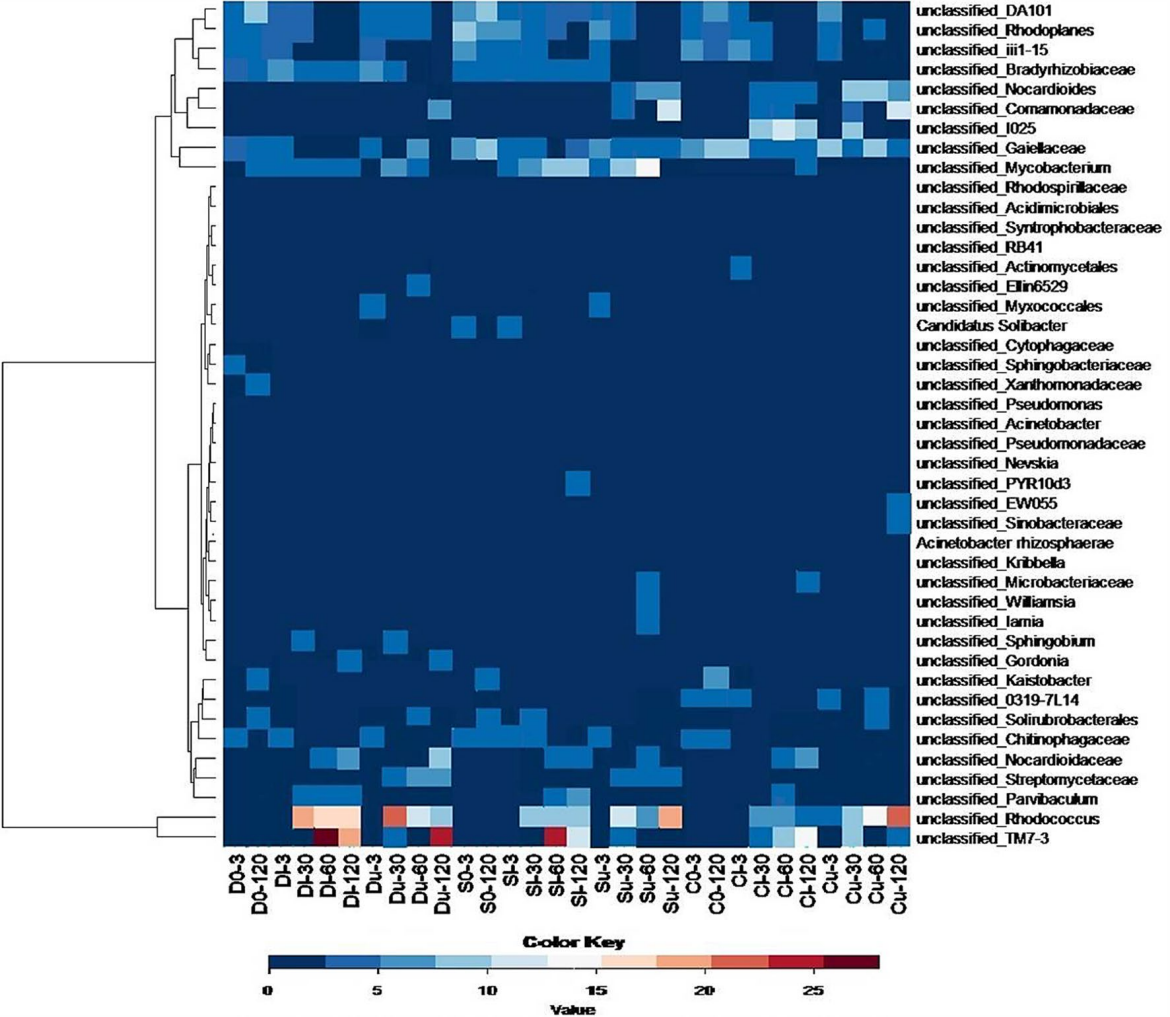
Heatmap of dominant bacterial genera (% relative sequence abundance ≥ 2). Changes are displayed on a relative scale with enrichment in red and depletion in blue.

At the phylum level, the bacterial composition in the polluted soils was similar to that in the unpolluted soils on the 3rd day of the experiment. Oil pollution led to an increased relative abundance of *Actinobacteria* and candidate *TM7* phylum (*Saccaribacteria*) and a decrease in *Bacteroidetes* on the 30th day of the experiment. A higher abundance of these phyla was observed until the end of the experiment. Relatively high relative abundances in oil-polluted soils were found for *Rhodococcus* sp. and representative of the candidate *TM7-3* class (up to 22% and 28%, respectively) (Fig. 4). The abundances of both OTUs did not exceed 1% in the unpolluted soil before the start of the experiment. Among the 20 most abundant OTUs in the polluted soils, half increased their relative abundance after pollution, while the other half was most abundant in the unpolluted soils. In addition to the two OTUs mentioned above, three other representatives of *Actinomycetales*, including two OTUs from the *Nocardiaceae* family, increased their relative abundance after pollution. These dominating OTUs caused shifts in bacterial composition at the phylum level, as shown in Fig. 4.

**Figure 5:**
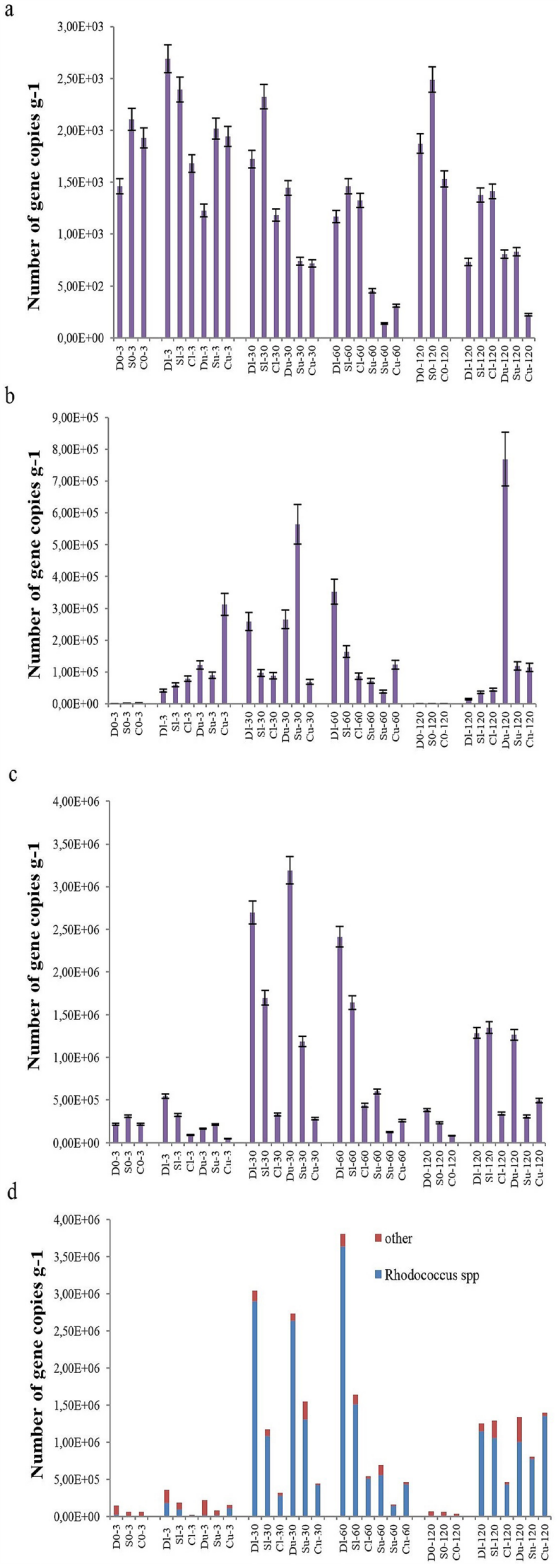
Gene copy numbers belonging to the alkI (a), alkII (b), and alkIII (c) groups and bacteria potentially carrying alkIII group genes (*Pseudomonas aeruginosa [alkB1]*, *Ps. fluorescens, Rhodococcus* spp. *[alkB1]*, *Burkholderia* spp. *[alkB]*, *Amycolatopsi*s spp. *[alkB]* (d)).

The number of alkII and alkIII gene copies increased after oil pollution in all the samples, while that of alkI did not depend on the presence, concentration, or duration of oil pollution (Fig. 5). All three gene groups encode alkane-degrading enzymes—alkI encodes enzymes attacking alkanes with 6–12 C atoms, and alkII and alkIII encode enzymes cleaving alkanes with longer chains^49–52^. A strong correlation was revealed between the alkIII gene copy number and abundance of taxa reported to carry these genes (*Pseudomonas aeruginosa [alkB1]*, *Ps. fluorescens, Rhodococcus* spp. *[alkB1]*, *Burkholderia* spp. *[alkB]*, *Amycolatopsi*s spp. *[alkB]*; R = 0.93). *Rhodococcus* sp. was the most abundant taxon possessing alkIII genes (up to 97% in polluted soils compared with 5–13% in the initial soils), and the correlation between its abundance and the alkIII group gene copy number was 0.92. Interestingly, the copy number of alkIII group genes in bacteria was high from 30–120 days of the experiment and was comparable to that of the control on day three. No correlation was found between the number of alkII group genes and their potential carriers—bacteria from the *Acinetobacter* genus.

**Figure 6:**
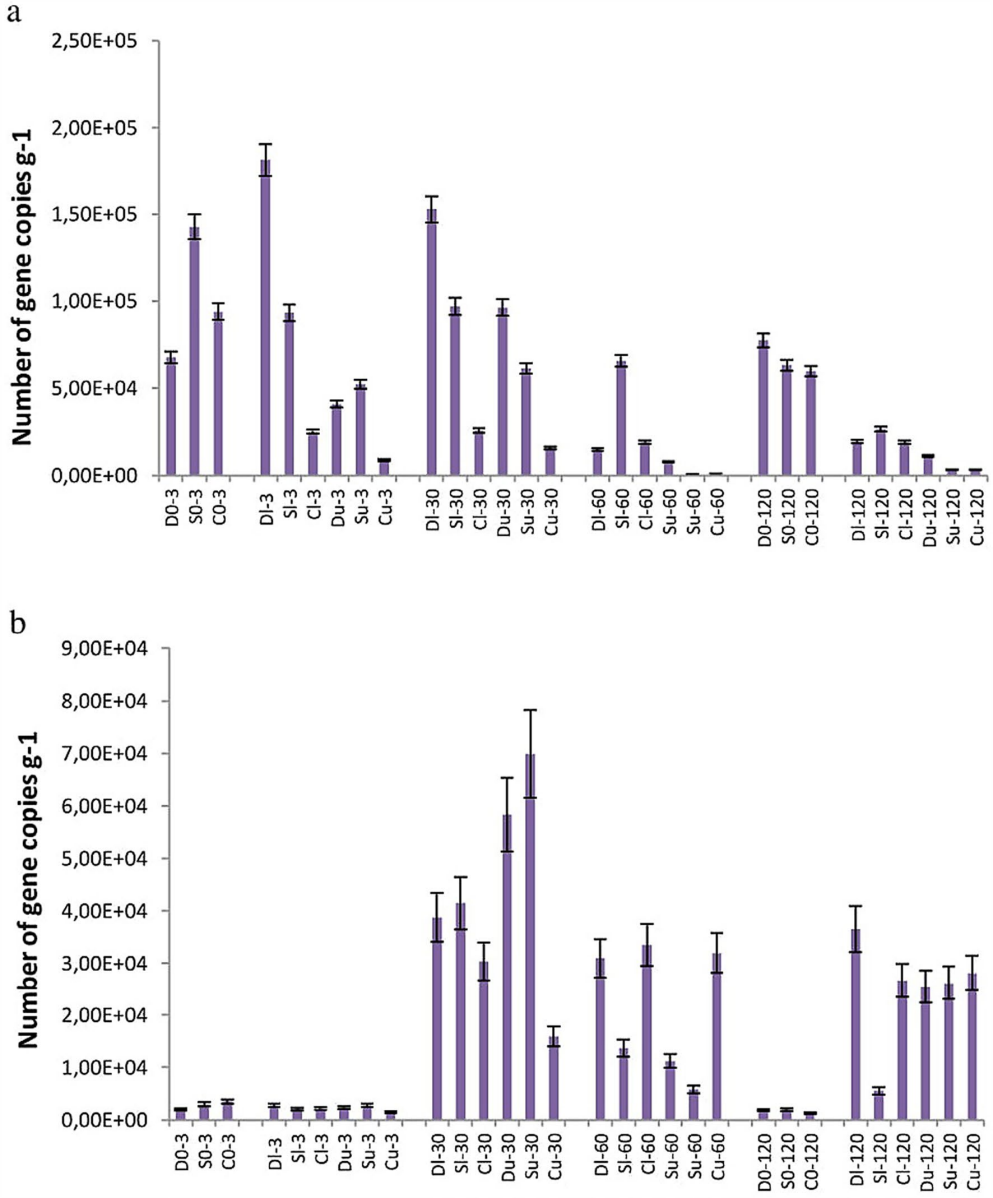
Gene copy numbers belonging to the groups GP-PAH (**a**) (*narAa, phdA/pdoA2, nidA/pdoA1,* and *nidA3/fadA1* described for *Rhodococcus, Mycobacterium, Nocardioides*, and *Terrabacter*) and GN-PAH (**b**) (*nahAc, nahA3, nagAc, ndoB, ndoC2, pahAc, pahA3, phnAc, phnA1, bphAc, bphA1, dntAc*, and *arhA1* described for *Pseudomonas, Ralstonia, Commamonas, Burkholderia, Sphingomonas, Alcaligenes,* and *Polaromonas*).

Furthermore, the copy number of genes encoding the ability of gram-negative and gram-positive bacteria capable of decomposing aromatic compounds was estimated (gene groups GN-PAH and GP-PAH, respectively; Fig. 6). Only genes belonging to the GN-PAH group (*nahAc, nahA3, nagAc, ndoB, ndoC2, pahAc, pahA3, phnAc, phnA1, bphAc, bphA1, dntAc*, and *arhA1* described for *Pseudomonas, Ralstonia, Commamonas, Burkholderia, Sphingomonas, Alcaligenes,* and *Polaromonas*)^53^ were dependent on the oil presence because their amount increased 5–30-fold 30 days after oil introduction and remained high until the end of the experiment in all three soil types. GP-PAH copy numbers did not change after oil pollution in soil. Moreover, no correlation was found between the GP-PAH gene copy number (narAa, phdA/pdoA2, nidA/pdoA1, and nidA3/fadA1 genes described for *Rhodococcus, Mycobacterium, Nocardioides*, and *Terrabacter*) and the abundance of dominating OTUs (*Rhodococcus* sp. and *TM7-3* class) that are known to be gram-positive (R = 0.05–0.09). Interestingly, neither GP-NAH nor the alkII and alkIII group gene numbers depended on the concentration of oil or type of soil, indicating that the strategies of the microbial communities were similar under the pressure of oil pollution in these samples.

### Fungal communities in oil-polluted soils

**Figure 7:**
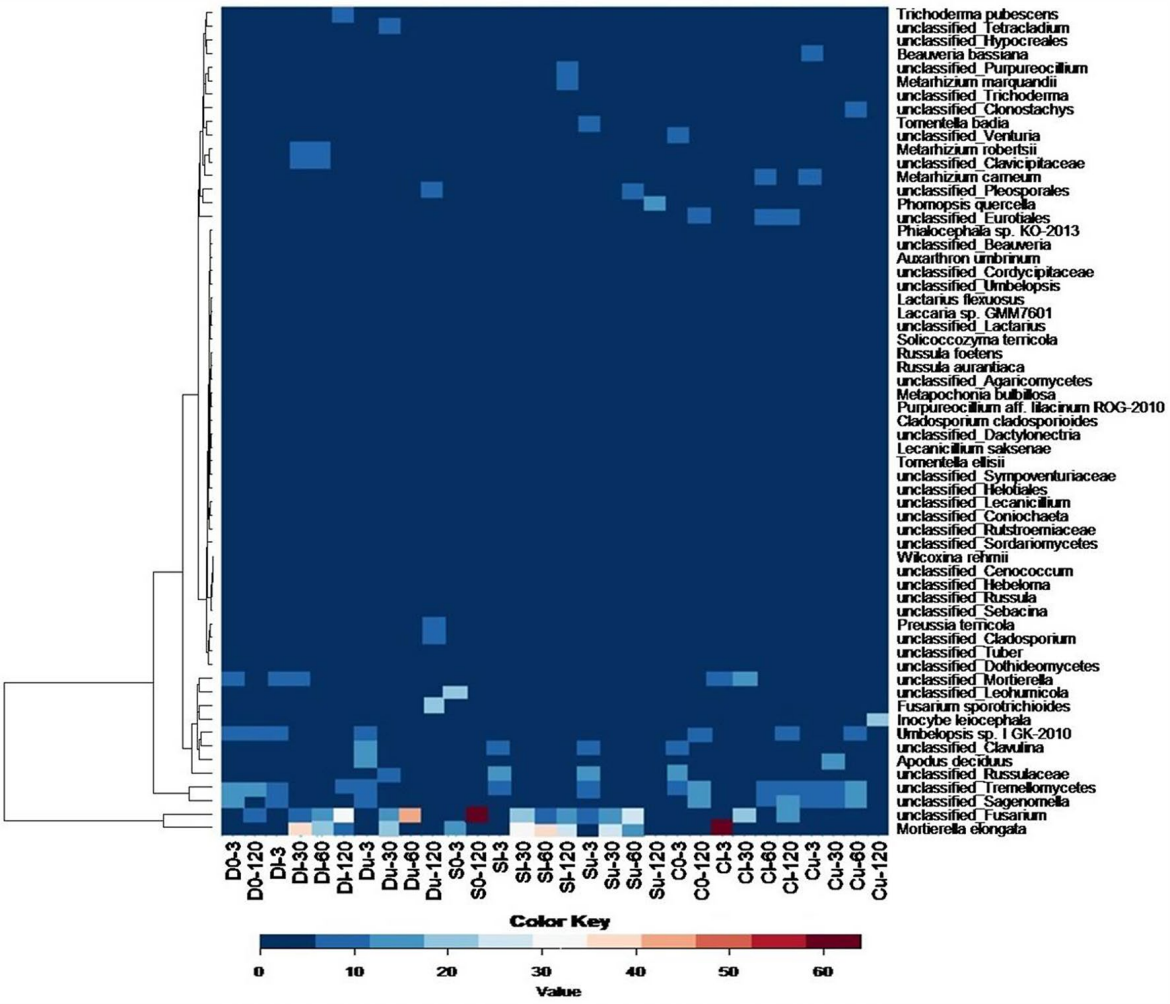
Heatmap of dominant fungal genera (% relative sequence abundance ≥ 2). Changes are displayed on a relative scale with enrichment in red and depletion in blue.

Because the composition of the fungal communities in the unpolluted soil was more diverse than that of the bacterial communities, oil pollution caused not only common but also soil type-specific shifts in the fungal community. Among the common trends, the codominance of *Ascomycota* and *Basidiomycota* on the 3rd day of the experiment, a high abundance of *Mycoromycota* along with decreased *Basiomycota* abundance on the 30th day of the experiment, and a gradual increase in *Ascomycota* abundance from the beginning until the end of the experiment were noted (Fig. 7). In all three soil types, the dominant OTUs maintained their levels after oil pollution, and new dominants occurred. Several fungal OTUs were ubiquitous in the polluted samples: *Fusarium* sp. and *Mortierella elongata*, which were dominant in the unpolluted soils. Genetically related OTUs of *Umbelopsis* sp. *I GK-2010,* and *Mortierella* sp. and representatives of the *Tremellomycetes* class became dominant in the polluted soils. *Sagenomella* sp.*, Apodus deciduous*, and a representative of the *Russulaceae* family are examples of the OTUs that were highly abundant once but were absent in the other types of polluted soils.

### Alpha- and beta-diversity of microorganisms in unpolluted and oil-polluted soils

**Figure 8:**
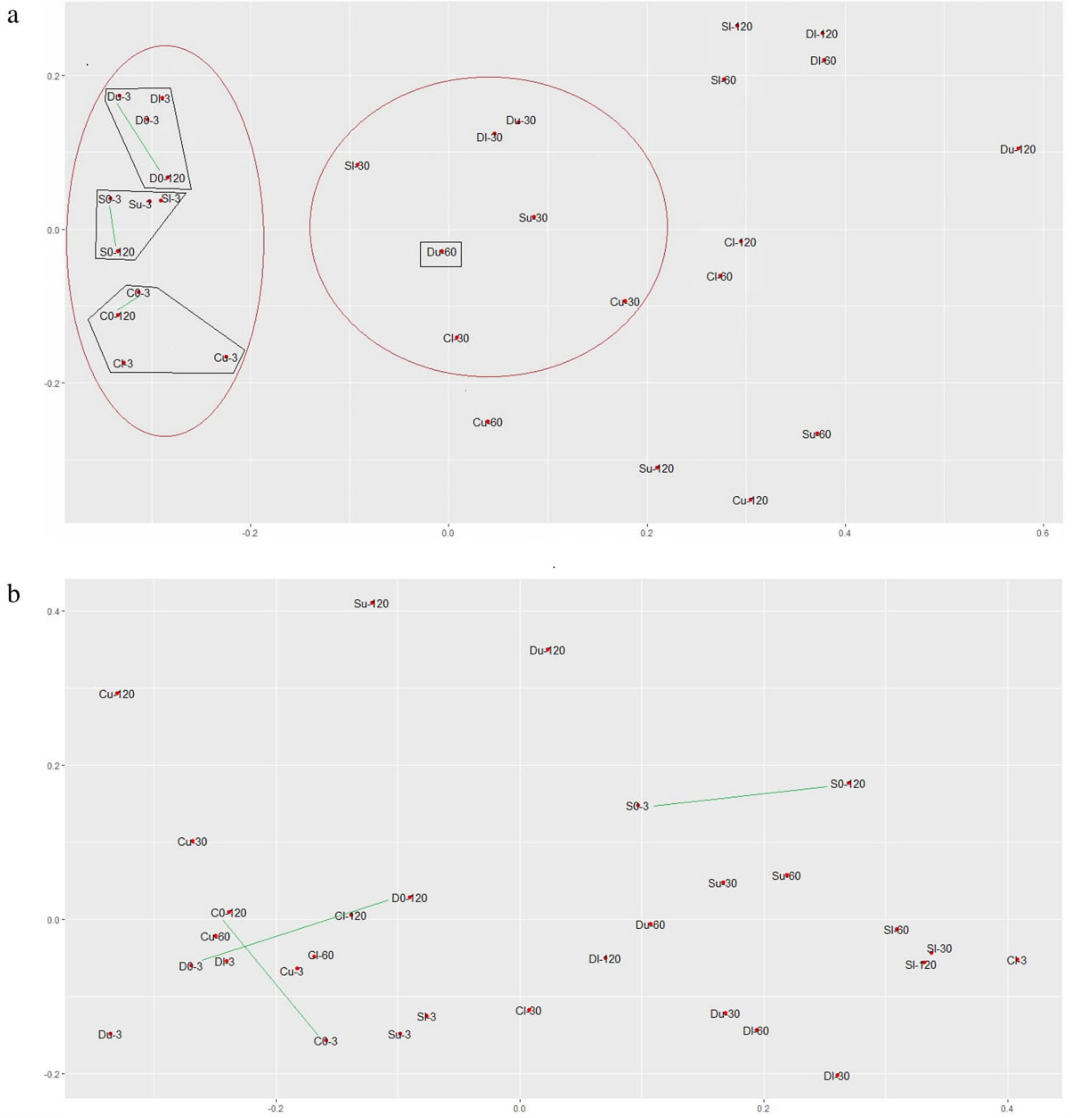
Boxplots illustrating alpha-diversity indexes (observed OTUs (**a**), evenness (**b**), and the Simpson (**c**) and Shannon (**d**) indexes) in bacterial (solid lines) and fungal (dashed lines) communities, where I—soils of various types (D, S, C); II—soils with different pollution levels (I, u); III—soils on the 0th, 30th, 60th and 120th days of the experiment. Median values and interquartile ranges are indicated in the plots.

The alpha diversity of the bacterial and fungal communities of unpolluted and oil-polluted soils was estimated using richness, evenness, the Simpson index, and the Shannon index. The results are presented in Fig. 8 in boxplots and grouped according to the soil type, petroleum concentration level, and day of experiment. Overall, the highest levels of indexes were registered for the bacterial community compared with fungal communities. The correlation index (R) between the alpha-diversity indexes for the two communities ranged from − 0.24 to 0.14.

Richness reflects the number of unique OTUs observed in the samples (Fig. 3, top). The highest richness for both the fungal and bacterial communities was observed in the C sample, and the fungal richness was approximately two–fourfold lower than the bacterial richness (Fig. 8a).The richness did not change among samples of different soil types and of different levels of oil pollution but decreased over time. This was true for both bacterial and fungal communities. In contrast with richness, evenness did not depend on soil type; in addition, for both bacterial and fungal communities, evenness was higher in the control soils than in the petroleum-polluted soils. The evenness ranged over time between 0.524 and 0.718 for bacteria and between 0.276 and 0.508 for fungi, and the trend of a decrease in evenness during the experiment coincided with that for richness. The Shannon index reflects the two factors of alpha diversity—abundance of the OTUs and evenness of the community^97^. In the unpolluted soils, the Shannon index ranged between 4.453 (S0 sample) and 4.692 (C0 sample) for bacteria and between 3.077 (D sample) and 3.244 (C sample) for fungi. Three days after oil pollution, the Shannon index values for bacterial communities were equal to those in the corresponding controls, independent of the oil concentration. The Shannon index was similar in soils of different types for both bacteria and fungi. This index was slightly higher for bacteria in the unpolluted soils than in the polluted soils, which was not the case for fungi. In line with the richness and evenness, the Shannon index decreased throughout the experiment in both communities. The Simpson index takes into account the number of OTUs present, as well as the relative abundance of each OTU. The greater the number of OTUs with high relative abundances is, the lower the Simpson index. In unpolluted soils, the Simpson index varied from 0.974 to 0.980 (D0 and C0 samples, respectively) for bacteria and from 0.905 (S0 sample) to 0.931 (C0 sample) for fungi. In oil-polluted soils, the Simpson index was found to be lower in D samples than in C and S samples for bacteria and lower in S samples than in C and D samples for fungi. The Simpson index of the bacterial community was higher in the unpolluted soils than in the polluted soils, which was not typical for the fungal community. In line with the three other diversity measures, the Simpson index tended to decrease during incubation for both bacterial and fungal communities.

**Figure 9:**
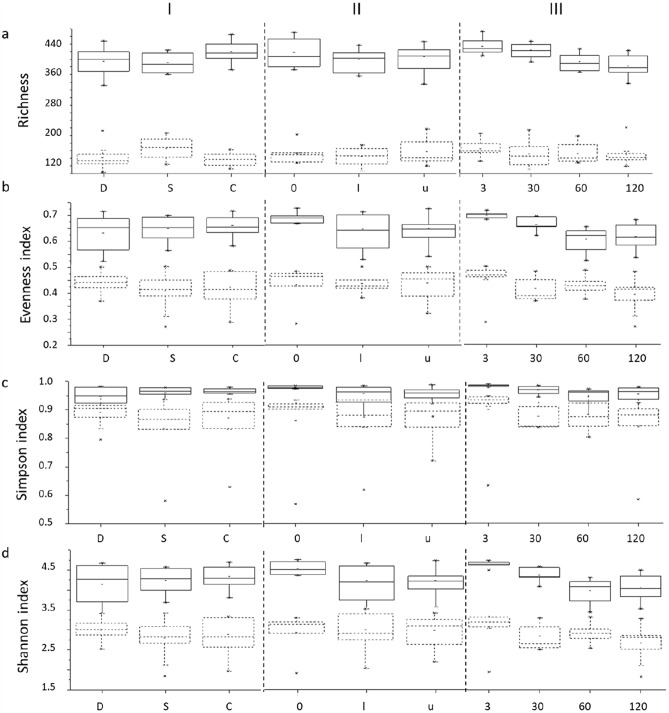
Beta diversity of bacterial (**a**) and fungal (**b**) communities in the initial and petroleum-polluted soils as revealed by NMDS analysis.

Beta diversity reflecting differences between the communities was estimated using the NMDS method (Fig. 9). In the NMDS plots, dots represent bacterial or fungal communities of different soil samples sampled on specific days (0, 3, 60 and 120), and the distance between them reflects similarities and dissimilarities between the communities: the closer the dots are, the more similar the communities are. As shown in Fig. 9a, dots representing bacterial communities were united into three groups, while the first group was divided into three subgroups: (i) subgroup a—D0-3, D0-120, Dl-3, Du-3, subgroup b—S0-3, S0-120, Sl-3, Su-3, subgroup c—C0-3, C0-120, Cl-3, Cu-3 communities; (ii) Dl-30, Du-30, Sl-30, Su-30, Cl-30, Cu-30, and Du-60 communities; and (iii) others. The first group comprised communities of unpolluted soils as well as those in soil shortly after pollution. The subgroups within this group united samples belonging to the specific soil types D, S, or C. The second group comprised communities of polluted soils sampled 30 days after pollution. This group was situated closer to the first group than to the third group, which comprised communities of polluted soils sampled 60 and 120 days after pollution (except for sample Du-60). Interestingly, the pairs of dots representing bacterial communities of unpolluted soils sampled with a 4-month difference were situated close to each other on the NMDS plots (D0-3 and D0-120, S0-3 and S0-120, S0-3 and S0-120). This was in contrast with dots representing the fungal communities. As shown in Fig. 9b, the distance between the corresponding dot pairs in unpolluted soils was comparable to that between the dots representing unpolluted and polluted samples. Overall, no clear trend associated with incubation time, type of soil, or level of pollution was observed for fungal communities.

## Discussion

Indigenous microbial communities can restore soil quality after petroleum pollution by biodegrading hydrocarbons. However, impactful biodegradation occurs at relatively low petroleum concentrations—up to 1–5% of soil weight—while biodegradation of higher hydrocarbon concentrations requires the implementation of additional methods of biostimulation or preliminary exclusion of the most polluted portion of soil from the remediation system^43–47^. Therefore, the microbial processes and composition of the microbial community have been well studied at low concentrations of petroleum but not at high concentrations. However, many polluted sites with a high petroleum content are in areas with intensive oil processing, resulting from accidents or illegal oil waste disposal^54,55^.

Because the initial concentration of petroleum in the soil in our experiment was high (from 6 to 25%), its content did not change significantly over time, as was previously found^45,46^. However, the fractional composition of petroleum was altered; alkanes and cycloalkanes were decomposed, whereas the proportion of heavier fractions increased. This outcome was evident for all three types of soils and both petroleum concentrations, indicating that differences in the organic carbon content, microbial abundance, composition, and activity of the microbial community do not play a role in alleviating extreme oil pollution in soils. Interestingly, the initial proportion of aromatics and aliphatics in soil was lower than that usually published for petroleum-polluted soils^56–58^. However, the data coincided with the composition of crude oil that was used in our incubation experiment (data not shown). This petroleum was obtained in the Republic of Tatarstan in Russia and was reported to be rich in heavy components^59,60^. The changes observed in the petroleum composition, together with the relatively high microbial abundance found in all the soil samples, indicate the microbial activity associated with petroleum biodegradation. Most likely, the microbial decomposition of petroleum hydrocarbons is less intense than that of soils with relatively low pollution levels. This microbial activity results in petroleum modification and, rarely, in its full mineralization.

Oil pollution led to a slight decrease in the number of bacterial gene copies in the soils, in particular, spiked with a high concentration of oil. This could be explained by the toxic effect of oil on soil microorganisms, as well as by indirect effects connected with changes in the physicochemical properties of the soil^22,61,62^. However, the number of fungal gene copies did not change significantly after oil pollution. This was most likely due to the ability of fungi to decompose hydrocarbons using nonspecific pathways normally used for the decomposition of lignin and consequent higher tolerance to hydrocarbons^22^.

The majority of bacterial communities in the initial soils were similar in terms of the OTU list and the dominating taxa. The most abundant OTUs from all three types of soils were *DAD101* sp. and representatives of the *Gaiellaceae* and *Chitinophagaceae* families, which are reported to constitute up to 10% of communities in undisturbed soils^63,64^. Bacteria from the *Bradyrhizobiaceae* family are nitrogen fixators and are typical for soils^65^. *Rhodoplanes* sp. and *Kaistobacter* sp. were previously described as dominant in the rhizosphere of agricultural crops, such as raps^66^. Bacteria from the *iii1-15* candidate order were found in soils of different geographic origins, including clean and oil-polluted soils^67–69^. Minor proportions of bacterial communities were unique in the soils, accounting for 6% (S) to 13% (C) of the total number of OTUs in the soils; additionally, the abundance of unique OTUs was low. According to the literature, soil microbial communities are one of the most diverse on Earth and usually possess a very rich microbial pool. The composition of the community at a specific time point depends highly on environmental conditions, such as pH, organic carbon content, redox potential, moisture, and phosphorous and nitrogen content, while fewer influencing factors include the soil texture, temperature, plant community composition, and other biotic factors^70,71^. The high similarity of the bacterial communities of the three different soil types found in our investigation may be explained by their close pH levels and organic carbon content, as well as by the close geographic location of the sampling sites (within several hundred km), which are reported to play an important role in bacterial community similarity^68^. However, in contrast to the bacterial communities, the fungal community compositions in the unpolluted soils differed from each other more significantly. Thus, 25% (D and C samples) to 43% (S sample) of fungal OTUs were unique, and only approximately 45% of the OTUs were ubiquitous. This outcome may be due to the stronger relationships between soil fungi and plant communities than between plants and bacteria in bulk soil^72–74^. At this point, it needs to be stressed that the sampling sites differed in plant cover. The complete list of plant species at the sampling sites was not recorded; however, the plant associations differed significantly visually, and the most abundant plant species were *Carex pilosa* for D soil, *Festuca pratensis* for S soil*,* and *Poa pratensis* and *Dactylis glomerata* for C soil. Fungal OTUs that were common for all three samples may be characterized as typical soil inhabitants^75–78^. Thus, the most abundant OTU in all three types of soils belonged to the *Fusarium* genus, which is widely spread in soils of all geographic zones and includes saprophytes and diverse plant pathogens^79,80^.

Oil pollution led to significant changes in the bacterial communities of all three soils. However, these changes were observed with some delay, despite the short life cycle of bacteria. This could be due to the slow rate of hydrocarbon decomposition, and bacteria have to adapt to the new carbon source and start growth^81^. The successions occurring after soil pollution with petroleum (at both concentrations) were similar, likely due to the similar bacterial community composition in the original unpolluted soils. Thus, oil pollution caused an increase in *Actinobacteria* abundance, mainly from the *Nocardiaceae* family (genera *Mycobacterium, Rhodococcus*, and *Nocardioides*). Such an increase agreed with the results presented previously^82–85^. One OTU from this phylum vastly increased its abundance between day three and day 30 of the experiment in all the soil type samples at both petroleum concentrations: *Rhodococcus* sp. *Rhodococcus* bacteria are reported to carry hydrocarbon-degrading genes from the alkIII group. Because of the ratio between the alkIII gene copy number and abundance, alkIII carriers (inclusive *Rhodococcus* sp.) did not grow after oil pollution, we suggest that the process of plasmid number increase in this OTU was absent or insignificant. Another OTU that was minor in unpolluted soils and became dominant in all polluted soils was that from the *TM7-3* class. Representatives of the *TM7* candidate phylum were previously described as potential hydrocarbon decomposers; however, because they have not been isolated and cultivated, little is known about their characteristic inclusive genes contained in their plasmids^86–88^. Because we did not find a correlation between the gene copy number of the alkII group and the abundance of *Acinetobacter* species reported to carry these genes, we suggest that OTUs from the *TM7-3* candidate class could contribute to the significant changes in this gene group. However, this suggestion should be additionally investigated. Another gene group copy number that increased after oil pollution was GN-PAH, which encodes a dioxygenase synthesis enzyme in gram-negative bacteria. Some gram-negative bacteria—e.g., those belonging to the *Pseudomonas* and *Acinetobacter* genera—are reported to be active hydrocarbon decomposers that can attack not only alkanes but also aromatic compounds^2,53^. Although the two main dominant bacteria in the oil-polluted soils described above were gram-positive, no increase in the GP-PAH copy number was observed after oil pollution in all three soils at either petroleum concentration. Thus, these two dominant OTUs possessed genes encoding monooxygenase but not the dioxygenase synthesis enzyme, indicating that they were presumably involved in alkane degradation in polluted soils of all three types. NMDS analysis of bacterial community composition confirmed the trends described above (Fig. 5). Indeed, the microbial composition of the unpolluted soils during 120 days of the experiment was close to that of the polluted soils of the same type sampled three days after pollution, independent of the petroleum concentration.

Differences existed between the bacterial communities of the unpolluted soils of three different types; however, they were less significant than those in the same sample analyzed at different dates (3rd, 30th, 60th, and 120th days of the experiment). According to the NMDS plot, the successions in bacterial communities in soils of three different types were unidirectional; thus, polluted samples analyzed on day 120 were closer to each other in terms of their bacterial community than to the corresponding unpolluted control samples. The alpha diversity in all the samples slowly decreased at the end of the experiment, likely due to the elimination of the initially minor OTUs and persistence of the major OTUs. To check this suggestion, logistic models were used that reflected the probability of zero abundance of OTUs in the later phases of the experiment, depending on their abundance in the initial soils and experiment duration (Table 2, Supplementary Tables S4, S5, S6). Indeed, it was demonstrated that the disappearance of minor species after petroleum pollution was the most significant factor in the decrease in alpha diversity in the communities, and this effect increased over the course of the experiment.

**Table 2:** Probability of eliminating bacterial OTUs depending on their initial abundance in unpolluted soils and on the experiment duration.

Variant	Dependence on the initial abundance of OTUs			Dependence on the duration of the experiment		
	Estimate	p-value	Significance	Estimate	p-value	Significance
Cl	0.002379	0.0334	*	− 23.028	< 2*10^–16^	***
Dl	0.005600	8.18*10^–7^	***	− 16.334	< 2*10^–16^	***
Sl	0.006140	1.11*10^–7^	***	− 13.556	2.92*10^–13^	***
Cu	0.005264	2.02*10^–6^	***	− 20.103	< 2*10^–16^	***
Du	0.007957	1.28*10^–12^	***	− 13.522	< 2*10^–16^	***
Su	0.005447	2.94*10^–6^	***	− 9.429	2.47*10^–13^	***

*Significant (p < 0.1).

***Highly significant (p < 0.001).

NMDS analysis of fungal communities (Fig. 9) confirmed the observation made based on the major phyla and dominant analysis presented above: the original communities of the three soils differed from each other more significantly than the bacterial ones, and the fungal communities of the unpolluted soils significantly altered over time. Most likely, these differences were the main reason for the absence of a common response of the fungal communities in the three different soils to petroleum pollution. However, the fungal OTUs that were found in the polluted soils showed some similarities. The *Fusarium* and *Mortierella* (*Umpelopsis*) species found in all three polluted soils could decompose lignin and cellulose and could attack petroleum compounds due to corresponding enzyme complexes. *Tremellomycetous* yeasts are universal representatives of terrestrial and aquatic ecosystems. OTUs belonging to this taxon were also found in all three polluted soils. *Tremellomycetes* are reported to be able to decompose hemicellulose and cellulose compounds, as well as degrade phenolic compounds. Other fungal OTUs that are dominant in polluted soils can be described as typical soil- and plant root-associated species^89–91^. It should be noted that in all three soils and at the two oil concentrations, the alpha diversity of fungal communities did not decrease, in contrast to that of bacterial communities, indicating that no strong domination or elimination of species occurred in the communities.

## Conclusion

Despite the high concentration of petroleum in soil and the insignificant rate of its decomposition, the soil microbial community not only sustained its quantity but also adapted to the new quality of the environment. The response of the bacterial and fungal communities showed common patterns for the three soil types investigated. Thus, bacteria possessing specific enzyme complexes can degrade hydrocarbons, as they drastically increase their ability to complete this degradation. We observed successions in the bacterial community structure, including alterations in dominant composition and decreased biodiversity. In fungal communities, the species that can degrade lignin, hemicellulose, and cellulose maintained their dominating positions because they possessed nonspecific enzymes enabling them to degrade aromatic compounds. Thus, changes in fungal communities were not as significant as those in bacterial communities. Interestingly, the bacterial communities of the three soils showed not only common patterns of response to petroleum pollution but also similar lists of dominant OTUs. This outcome may be due to the significant overlap of the bacterial pools in the three soils before pollution.

We demonstrated that even soils heavily polluted by petroleum (up to 25% petroleum content) have the potential for biological restoration: the counts of bacteria and fungi are comparable with those in unpolluted soils, and the communities are “hydrocarbon degradation”-oriented in terms of their OTU composition. This suggests that bioremediation strategies may be efficiently developed and implemented for such types of polluted soils.

## Methods

### Experimental design

Three samples of different soil types determined according to the soil map and confirmed by own investigations were taken in different areas of the Republic of Tatarstan in Russia (D—umbric albeluvisol, latitude: 55°48′59.77″ N, longitude: 49°20′24.51″ E; S—albic luvisol, latitude: 55°30′12.58″ N, longitude: 49°51′03.03″ E; C—vermic chernozem, latitude: 55°17′40.28″ N, longitude: 50°06′53.99″ E). The soil samples were preincubated at room temperature and 60% of field water capacity for 7 days. Each sample was then divided into nine subsamples with a mass of 10 kg each and was placed into a plastic container. Urals crude oil sampled in Tatarstan (Russia) was added to three subsamples at 60 g kg^−1^ soil and to another three subsamples at 250 g kg^−1^ soil (samples l and u (lower and upper level of pollution), respectively). Three subsamples for each soil type were used as controls (zero samples). Thus, nine variants of soil samples were used in this study—D0, Dl, Du, S0, Sl, Su, C0, Cl, and Cu —and each variant was incubated in three replicates in glass incubators with the maintenance of a constant air humidity at room temperature over 120 days. Once a week, water was added to the soil to maintain 60% of field water capacity.

### Chemical properties of petroleum and soil samples

Extraction of the total hydrocarbon content from the soil was carried out by selective extraction of chloroform bitumoid in two stages (the cold and second hot extractions were performed in a Soxhlet apparatus)^92^. The total petroleum content was estimated using a gravimetric method. The fractions of the aliphatic and aromatic hydrocarbons, resins, and asphaltenes were separated using the Marcusson method^93^. Briefly, the extracted TPH was dissolved in chloroform and loaded onto the column. Successive elution was performed using petroleum-ether, benzene, and chloroform to obtain the aliphatic, aromatic, and polar hydrocarbon fractions, respectively. The fractions were then determined gravimetrically after solvent evaporation.

The fraction of saturated and aromatic hydrocarbons was analyzed using gas chromatography–mass spectrometry (GC–MS). The analysis was carried out using a Chromatek-Crystal 5000 gas chromatography mass spectrometer in the SCAN mode (Chromatek, Russia). Hydrocarbon separation was carried out using a capillary column 30 m long and 0.32 mm in diameter with a PE-XLB phase. The GC oven was programmed using the following protocol: from 100 to 150 °C at 12.5 °C min^−1^, from 150 to 300 °C at 3 °C min^−1^, and held at 300 °C for 14 min; helium with a velocity of 2 ml min^−1^ was used as the carrier gas.

### DNA extraction and amplification

Total genomic DNA of the samples was extracted using the FastDNA SPIN Kit for Soil (Bio101, Qbiogene, Germany) according to the manufacturer’s instructions. In all cases, DNA extraction was performed in triplicate, and the samples were stored at − 20 °C or analyzed immediately.

The abundance of the specific genes (Table 3) in each sample was quantified by qPCR. For bacteria, 16S 341f. (5′-CCTACGGGAGGCAGCAG-3′) and 534r (5′-ATTACCGCGGCTGCTGG-3′) primers were used^94^. In the fungal assay, FungiQuant-F (5′-GGRAAACTCACCAGGTCCAG -3′) and FungiQuant-R (5′-GSWCTATCCCCAKCACGA-3′) primers were used^95^. PCR amplification was performed using the CFX96 Touch Real-Time PCR Detection System (Bio-Rad, Munich, Germany) according to the following protocol: 15 min at 95 °C, followed by 39 cycles at 95 °C for 30 s, 30 s at Tm and 30 s at 72 °C. PCR was carried out using 0.1 U µl^−1^ of SynTaq Polymerase, 1 × SYBR Green Buffer, 2.5 mM MgCl_2_, 200 µM of each dNTP, 0.2 µM of each primer and 1 µl of DNA template. Standard curves were generated for bacteria using serial DNA dilutions from *Bacillus pumilus* and *Penicillium chrysogenum.* The number of copies of the target gene was calculated directly from the concentration of the extracted plasmid DNA. Ten-fold serial dilutions of a known number of copies of plasmid DNA were subjected to real-time PCR in five replicates to obtain an external standard curve. The DNA concentration was measured using a Qubit 3.0 Fluorometer (Thermo Fisher Scientific, USA) and Quant-iT dsDNA High-Sensitivity Assay Kit (Thermo Fisher, USA). Three replicates for each sample were used for qPCR analysis. The efficiency of qPCR assays was 94%, and the R2 value was greater than 0.99.

**Table 3:** Primers used in the work.

Group of genes	Primer sequence	Tm	References
alk I	CATAATAAAGGGCATCACCGT GATTTCATTCTCGAAACTCCAAAC	55 °C	(Kohno, 2002)^96^
alk II	GAGACAAATCGTCTAAAACGTAA TTGTTATTATTCCAACTATGCTC	55 °C	
alk III	TCGAGCACATCCGCGGCCACCA CCGTAGTGCTCGACGTAGTT	55 °C	
GP-PAH	CGGCGCCGACAAYTTYGTNGG GGGGAACACGGTGCCRTGDATRAA	56 °C	(Cebron, 2008)^97^
GN-PAH	GAGATGCATACCACGTKGGTTGGA AGCTGTTGTTCGGGAAGAYWGTGCMGTT	57 °C	

### MiSeq Illumina sequencing

In oil-contaminated soils sampled on the 3rd, 30th, 60th and 120th days of the experiment, the structure of the bacterial community was determined by 16S rRNA sequencing using Illumina MiSeq. Noncontaminated soils analyzed on the 3rd and 120th days were used as controls.

Preparation of the libraries was performed in accordance with the 16S Metagenomic Sequencing Library Preparation Protocol recommended for Illumina MiSeq. The first round of amplification of the V3-V4 region of the 16sRNA gene was performed using the DNA Engine Tetrad 2 cycler (BioRad, Germany) with specific primers A (TCGTCGGCAGCGTCAGATGTGTATAAGAGACAGCCTACGGGNGGCWGCAG) and B (GTCTCGTGGGCTCGGAGATGTGTATAAGAGACAGGACTACHVGGGTATCTAATCC). Amplification of the ITS2 region was performed using the ITS86F-ITS4 primers. PCR amplification was carried out according to the following protocol: initial denaturation for 3 min at 95 °C, 27 cycles of 30 s at 95 °C, 30 s at 55 °C, and 30 s at 72 °C; and a final extension for 5 min at 72 °C. Further purification of amplicons was performed using the Agencourt AMPure XP purification kit (Beckman Coulter, USA). The second round of amplification was performed for double indexing of samples using the same cycle parameters.

The library obtained was validated using the Bioanalyzer 2100 system and the Agilent DNA 1000 Kit (Agilent, USA) and was quantified using the Qubit 3.0 Fluorometer (Thermo Fisher Scientific, USA) and Quant-iT dsDNA High-Sensitivity Assay Kit (Thermo Fisher, USA). Purified amplicons were pooled at equal concentration. Further preparation of samples and sequencing were performed using MiSeq Reagent Kit v2 (300 cycles) and MiSeq-device (Illumina, USA) according to the manufacturer’s protocol.

### Sequence analysis

After sequencing, the previously added adapter sequence was removed, and then the samples were evaluated using the index sequence and Illumina BaseSpace software.

16S rRNA sequencing data were analyzed using the Quantitative Insights Into Microbial Ecology (QIIME2) platform, version 2019.4 software^98^. The number of raw reads ranged between 22,641 and 85,460 for 16S rRNA amplicons (Supplementary Table S7). Data was normalized by QIIME using rarefaction by the smallest reads file. After the quality filtering step (Q < 20), chimeras were removed using the usearch61 algorithm. Operational taxonomic unit (OTU) clustering against the Greengenes database (2013-08 release) was performed using the implemented USEARCH pipeline with a 97% sequence identity threshold. Only OTUs represented by at least five reads were kept. Taxonomic classification was performed using the implemented RDP classifier with PyNAST^99^.

Quality control of the readings of the obtained ITS2 sequences was performed using cutadapt v2.7^100^. The pair-end readings were combined using FLASH v1.2^101^, and chimeras were removed using RDP Tools^102^. The number of raw reads ranged between 20,140 and 37,418 for ITS2 amplicons (Supplementary Table S7). Data was normalized using RDP Tools. Taxonomic classification was carried out according to the ITS2 database^103^ using RDP Tools v2.11, with a sequence identity threshold of 97%.

### Statistical analysis

Statistical analyses were performed using the R Statistical Software package (R 3.6.1 version)^104^. Hierarchical clustering analysis of microbial profiles was performed with the Euclidean distance measure and average clustering algorithm to visualize the results in a heatmap. The measures of alpha-diversity calculated for each community were unique taxa richness (numbers of OTUs), the Shannon–Weaver index^105^, Simpson index^106^, and Shannon Evenness^105^. The relative similarities of the bacterial and fungal communities revealed in the two composts were verified using nonmetric multidimensional scaling (NMDS) based on the Bray–Curtis coefficient according to a procedure presented by Clarke (1993)^107,108^.

## Supplementary Information

Supplementary Information.
